# Personalising your health: an EU imperative

**DOI:** 10.3332/ecancer.2017.ed68

**Published:** 2017-07-11

**Authors:** Denis Horgan

**Affiliations:** EAPM, Avenue de l’Armee, Legerlaan 10, 1040 Brussels, Belgium

**Keywords:** policy, personalised medicine, EU Member State

## Abstract

In the fast-moving world of personalised medicine there are many issues and barriers—not least when it comes to getting novel drugs and treatments swiftly to where they are really needed. Slow bench-to-bedside rates do not help the 500 million potential patients across the EU’s current 28 Member States, and the timings are affected by several elements during the development and licensing phases. As we all know, personalised medicine starts with the patient. It holds huge potential for improving the health of many patients and ensuring better outcomes for health systems’ efficiency and transparency.

## Overview

Personalised medicine offers the promise of seeing healthcare move away from ‘trial-and-error’ therapies to evidence-based individual ones, removing the ‘one-size-fits-all’ philosophy.

It starts with the patients, and holds huge potential for improving the health of many of them.

However, its integration into clinical practice and daily care is proving difficult given the many barriers and challenges to timely access to targeted healthcare that still exist [1].

As one of its core values, the EU holds to the ideal of equality and a clear way to measure success in this area is through the well-being of all of its (currently) 500 million citizens.

Health is a key aspect in well-being and politicians know that, if you ask any citizen, health and healthcare will be high on their agenda—and, as we live longer, that will become more the case, rather than less.

It is incumbent upon all stakeholders in the realm of healthcare—and especially the policymakers and legislators—to ensure that every citizen of Europe has the same rights and access to the same high quality care as his neighbour [2].

## Relevance of policy in shaping personalised medicine

Personalised medicine relies on new technologies (genomics etc), plus Big Data and better-targeted clinical trials in order to deliver the right treatment to the right patient at the right time.

Policymakers have a vital role to play if the potential of personalised medicine is to be fully realised [3].

Areas include the need for more promotion for research, education programmes, incentives for innovators and better regulation, plus commonly agreed (and widely implemented) best practices.

Much of healthcare remains a Member State competence, therefore the EU cannot act alone. Member States have to adapt, take a look at their HTA/approval procedures and collaborate much more other EU countries in many spheres, including the sharing of knowledge and research, and the pooling of Big Data [4].

It is evident that the EU, in tandem with Member States, needs to ensure the proper transposition of its legislation and policies regulation at national level.

## The core needs for EU and Member States policymakers to address

**Innovation** is key to progress and, currently, there is a lack of **incentives**.

There is a surfeit of incentives to promote investment in developing diagnostics. Coordinated timing in reimbursement and approval of a companion diagnostic is essential. Also, the transferring of technology and public health assessments differ from state-to-state [5].

**Translation of research** affects both treatment once a disease has been discovered and, importantly, prevention—with all the cost savings that go along with the latter, not to mention continuation of the best quality of life.

Translating cutting-edge research effectively in the arena of personalised medicine will save lives and lower costs.

Policymakers need to be aware that Europe must develop **education and training** for healthcare professionals (HCPs) whose disciplines are essential to the successful development of personalised medicine. Clearly, there is a knowledge gap that has to be addressed.

What is required is a long-term approach to education in order that all HCPs in close contact with patients or their patients’ families need to be up-to-date with the current aspects of personalised medicine.

From a **policymaking and regulatory** perspective, Europe has reached a point in the healthcare of its citizens when clear, harmonised rules and guidelines need to be put in place across the entire EU [6].

The EU cannot, as it stands, assume responsibility for all healthcare systems across Europe, but it can certainly recommend it in the areas over which it does not have legal competence [7].

But first its policymakers must listen and thoroughly understand all of the issues affecting access.

## Relevance of personalised medicine in shaping policy

Advocates of personalised medicine have engaged with various rotating Presidencies of the EU in recent years to put forward the views and ‘asks’ of their expert members, which of course include patients.

For example, the Luxembourg Presidency held a Summer 2015 conference on improving access to personalised medicine which was a key influence in that country’s landmark Council Conclusions [8].

While Council Conclusions can only make recommendations to Member States, the potential for influence is significant once these conclusions are adopted by the Member States’ health ministers.

Engagement on the topic of personalised medicine with the Parliament, Commission and Council has been key to several major pieces of legislation in recent years, including the General Data Protection Regulation, the Clinical Trials Regulation and legislation on In Vitro Diagnostics.

As well as regular interaction with the Commission, there are many MEPs who have a strong interest and belief in the advancement of personalised medicine.

Patients are also doing a great deal to help make their own voices heard, are are more knowledgable than ever before.

On top of this, personalised medicine groups are starting to form across Europe and more will follow.

Media articles on personalised medicine are being seen more and more, which helps to raise awareness among other journalists and the general public.

## Conclusions

It is clear to the EU and Member States that the current situation in healthcare systems across Europe is unsustainable. Solutions need to be found, and quickly.

There needs to be a paradigm shift from the one-size-fits all model to a smarter, more sustainable way of operating and, with its targeted therapies and use of fast-moving science, personalised medicine has a key role [10].

Personalised medicine has now found its way into EU and Member State policymakers’ mindsets and has had a profound effect in key areas of recent policy and legislation. This will become more and more the case, in the short-, medium- and long-term.

EAPM’s co-chair Gordon McVie, the founder of *e*cancer, has recently worked with Pfizer to produce an exceptionally useful video on health economics, which you can view here: http://ecancer.org/video/6032/-pfizer--the-challenge-of-approving-add-on-therapies-for-patients-with-metastatic-cancer.php.

As discussed, there are different standards of healthcare in different countries, different price structures in many of them, and problems in affordability when it comes to cross-border access for patients [11].

More money is not necessarily the primary driver. Europe needs to think along the lines of a new legislative and investment framework.

If personalised medicine is to be in line with the EU and Member State principle of universal and equal access to high-quality healthcare, then clearly it must be made available to many more citizens than it is now.

Regulators and policy makers at every level have a major role to play.
